# Phytochemical Profiles and Anticancer Activity of the Three *Prosopis* Species Growing in Qatar Against Lung and Ovarian Cancer Cells

**DOI:** 10.3390/molecules31193464

**Published:** 2026-09-29

**Authors:** Salma M. S. Ahmad, Zainab Farooq, Maysa Z. Masalmeh, Iman Saleh, Fatiha Seglab, Layla Al-Mansoori, Mohammed H. Abu-Dieyeh, Abdullah A. Shaito

**Affiliations:** 1 Biomedical Research Center (BRC), QU Health, Qatar University, Doha P.O. Box 2713, Qatar; sa1701249@qu.edu.qa (S.M.S.A.); almansouri@qu.edu.qa (L.A.-M.); 2 Department of Biomedical Sciences, College of Health Sciences, QU Health, Qatar University, Doha P.O. Box 2713, Qatar; 3 Biological Science Program, Department of Biological and Environmental Sciences, College of Art and Science, Qatar University, Doha P.O. Box 2713, Qatar; imanesaleh@qu.edu.qa; 4 College of Medicine, QU Health, Qatar University, Doha P.O. Box 2713, Qatar

**Keywords:** *P. farcta*, *P. cineraria*, *P. juliflora*, A549, SK-OV-3, apoptosis, proliferation, cell migration

## Abstract

Lung and ovarian cancers account for substantial cancer morbidity and mortality worldwide. Plant-derived natural products represent promising alternatives in cancer therapy because of their structural diversity and ability to target multiple signaling pathways. *Prosopis* species are ethnomedicinal plants traditionally known for their antimicrobial, anti-inflammatory, and antioxidant activities; however, their anticancer potential remains poorly explored. This study evaluates the anticancer properties of extracts of *Prosopis cineraria*, *Prosopis farcta*, and *Prosopis juliflora*, the *Prosopis* species growing in Qatar, against human lung (A549) and ovarian (SK-OV-3) cancer cells. Gas chromatography–mass spectrometry (GC–MS) was used to characterize their phytochemical composition. The cytotoxicity and anti-migratory activity of the three extracts were evaluated. Western blot analysis was performed to investigate the molecular effects of the extracts on proliferation- and apoptosis-related proteins. GC–MS profiling identified diverse low-molecular-weight bioactive compounds across the extracts, including several compounds detected only in *P. juliflora*, which exhibited the most potent cytotoxicity toward A549 and SK-OV-3 cells. All three extracts inhibited cancer cell migration, with *P. juliflora* showing the strongest effect. Furthermore, *P. juliflora* reduced the expression of proliferation and survival markers and increased the expression of pro-apoptotic proteins. These findings identify *P. juliflora* as the most active of the three tested *Prosopis* extracts and support further investigation of its in vitro anticancer potential.

## 1. Introduction

Cancer remains a major global health burden, with nearly 20 million new cases and 9.7 million deaths reported worldwide in 2022 [1]. Lung cancer is the most commonly diagnosed cancer and the leading cause of cancer mortality, accounting for approximately 2.5 million new cases and 1.8 million deaths in 2022. Despite advances in surgery, chemotherapy, radiotherapy, immunotherapy, and targeted therapy, clinical outcomes remain poor, with an overall 5-year survival rate of approximately 19% and survival as low as 2–3% in extensive-stage small cell lung cancer [1,2,3]. Ovarian cancer, although less prevalent, is the most lethal gynecologic malignancy and remains a major cause of cancer-related mortality among women [1,4]. Standard treatment consists primarily of cytoreductive surgery followed by platinum-based chemotherapy, yet most patients are diagnosed at advanced stages, and approximately 80% ultimately experience disease recurrence [1,5,6]. Although targeted therapies, including bevacizumab and poly(ADP-ribose) polymerase (PARP) inhibitors, have improved progression-free survival in selected patient populations, durable disease control remains challenging [7].

Despite advancements in cancer treatment, late-stage diagnosis, treatment-associated toxicity, tumor heterogeneity, suboptimal treatment responses, the development of drug resistance, and disease relapse continue to limit clinical outcomes [2,8]. These challenges underscore the need to identify therapeutically active compounds with novel or complementary mechanisms of action. Natural products, particularly plant-derived secondary metabolites, remain valuable sources of anticancer agents because of their substantial chemical diversity, capacity to modulate multiple cancer-associated pathways through distinct mechanisms, potential for selective toxicity toward cancer cells, broad availability, relative abundance, and relatively low production costs [9,10,11,12].

The genus *Prosopis* (Fabaceae) comprises approximately 45 species of drought-tolerant shrubs and trees distributed across Africa, Asia, and the Americas [13,14]. *Prosopis* species extracts, fractions, and secondary metabolites have demonstrated numerous biological activities, including antimicrobial, anti-inflammatory, antidiabetic, antioxidant, and cytotoxic properties, largely attributed to a rich and diverse phytochemical composition of secondary metabolites dominated by tannins, phenolic acids, flavonoids, c-glycosyl flavones (such as vitexin, isovitexin, vicenin II), quinones, and alkaloids [13,15,16]. Importantly, *Prosopis* species have demonstrated numerous cytotoxic, antiproliferative, pro-apoptotic, and tumor-suppressive activities in experimental cancer models, supporting the genus as a potential source of anticancer bioactive compounds [17,18].

*Prosopis cineraria* (L.) Druce, *P. farcta* (Banks & Sol.) J.F. Macbr., and *P. juliflora* (Sw.) DC. [synonym. *Neltuma juliflora* (Sw.) Raf.] are the three *Prosopis* species growing in Qatar. *P. cineraria* contains phenolic acids, flavonoids, tannins, glycosides, sterols, and specialized metabolites, including chlorogenic acid, caffeic acid, luteolin, spicigerin, prosogerins, and β-sitosterol [15,16,19]. Its anticancer potential is supported by inhibition of MOLT-4 leukemia [20], HepG2 hepatocellular carcinoma, U87MG glioblastoma [21], MCF-7 breast cancer [22,23], and Huh-7 hepatocellular carcinoma cells [24]. Antitumor effects have also been reported in Ehrlich ascites carcinoma and chemically induced hepatocarcinogenesis models [17,25].

*P. farcta* contains phenolic acids, flavonoids, stilbenes, coumarins, alkaloids, and other low-molecular-weight constituents [15,26]. Although comparatively less studied, its extracts have shown cytotoxic activity against HepG2 liver, HeLa cervical, PC3 prostate, and MCF-7 breast cancer cells [26]. In HT-29 colorectal cancer cells, *P. farcta* methanolic extract induced mitochondrial dysfunction and reactive oxygen species (ROS)-mediated apoptosis [27].

*P. juliflora* (Al Ghuweif or Mesquite) exhibits diverse biological activities, including antioxidant, antidiabetic, and antimicrobial effects [15,28,29], supported by a rich phytochemical profile comprising phenolic acids, flavonoids such as mesquitol, tannins, glycosides, and piperidine alkaloids, including juliprosopine, julifloricine, and juliprosinene [30,31]. Growing evidence highlights the anticancer potential of *P. juliflora*. Its extracts have been reported to induce apoptosis and suppress proliferation in MCF-7 breast, HepG2 liver, and LS-174T colorectal cancer cells [32,33]. Additional studies have demonstrated apoptosis and cell-cycle arrest in breast cancer models, suppression of epithelial-to-mesenchymal transition, migration, invasion, angiogenesis, and stemness-associated markers in B16F10 melanoma cells [34], and caspase-3-mediated apoptosis in LNCaP prostate cancer cells [35].

Collectively, these studies establish a rationale for investigating the anticancer potential of *P. cineraria*, *P. farcta*, and *P. juliflora*. However, the comparative anticancer activity of these three species in lung and ovarian adenocarcinoma models, and its association with their phytochemical profiles, remains poorly characterized. To our knowledge, this is the first comparative study to integrate phytochemical profiling with functional evaluation of these three species in human lung and ovarian cancer models. A549 and SK-OV-3 cells were selected as established human lung and ovarian adenocarcinoma models, respectively, to evaluate the activity of the *Prosopis* extracts across two biologically distinct cancer models, consistent with the use of these human tumor cell lines for preclinical anticancer screening [36], while human neonatal dermal fibroblasts (HDFn) were included as a non-malignant comparator to assess differential cytotoxicity and extract selectivity [37]. Accordingly, we characterized the low-molecular-weight constituents of the three extracts using gas chromatography–mass spectrometry (GC–MS) and evaluated their antiproliferative, pro-apoptotic, and anti-migratory activities in A549 and SK-OV-3 cells.

## 2. Results

### 2.1. Identification of Compounds of P. cineraria, P. farcta, and P. juliflora Leaf Extracts by GC–MS

To establish a comprehensive profile of the low molecular weight compounds of *P. cineraria, P. farcta*, and *P. juliflora* leaf extracts, GC–MS analysis was conducted, unveiling 20, 7, and 27 compounds, respectively (Figure 1 and Table 1). The metabolites, listed in Table 1 and arranged by retention time, were identified by comparing their mass spectral fragmentation patterns with those of known compounds in the NIST library. The identified compounds encompass various classes of secondary metabolites, including terpenes, terpenoids, sterols, long chain alkanes, alcohols, epoxides, and ketones, α-tocopherol (vitamin E class antioxidant), chanoclavine (an alkaloid), and astaxanthin (a carotenoid). Some of the compounds are common to all three plants or among two of the plants. Others are species-specific; for example, betulin, 12-oleanen-3-yl acetate, spathulenol, and lanosterol are exclusive to *P. cineraria*. Astaxanthin is unique to *P. farcta*, and compounds such as humulene, α-curcumene, isopulegol, octacosane, and limonene oxide are unique to *P. juliflora* (Table 1).

Many of these phytochemicals are recognized for their diverse biological activities, including antioxidant, anti-inflammatory, antimicrobial, and cytotoxic properties [38,39,40,41,42,43,44], underscoring the therapeutic potential of these *Prosopis species* and supporting their traditional medicinal uses.

### 2.2. P. juliflora Leaf Extract Reduces the Viability of Human Ovarian and Lung Cancer Cells

To examine the cytotoxic effects of *P. cineraria*, *P. farcta*, and *P. juliflora* extracts on A549 lung cancer cells, SK-OV-3 ovarian cancer cells, and HDFn normal neonatal fibroblasts, Alamar Blue cell viability assay was performed (Figure 2). *P. cineraria* did not significantly affect the viability of HDFn, A549, or SK-OV-3 cells at the tested concentrations (more than 95% viability) (Figure 2A). Similarly, *P. farcta* extract, at the tested concentrations, did not exhibit significant cytotoxic effects in HDFn or A549 cells, and only a modest, non-significant reduction in viability in SK-OV-3 cells (Figure 2B). These findings suggest that *P. cineraria* and *P. farcta* are not cytotoxic at the tested concentrations in the three cell lines. In contrast, *P. juliflora* extract showed marked reduction in cell viability, particularly against cancer cells (Figure 2C). A549 cells were markedly sensitive to *P. juliflora* treatment, displaying significant reductions in viability at all the examined concentrations, starting at 25 µg/mL (53% reduction in cell viability). SK-OV-3 cells also exhibited a strong dose-dependent decrease in viability at all the tested concentrations, starting at 25 µg/mL (72% reduction in cell viability). Indeed, *P. juliflora* extracts potently reduced the viability of A549 and SK-OV-3 cells with IC50 values of 42.2 ± 2.55 and 49.8 ± 5.34 µg/mL, respectively. Moreover, the *P. juliflora* extract caused a decrease in HDFn cell viability with a calculated IC50 of 128 ± 5.6 µg/mL; however, this decrease was only significant at the highest tested concentration (75 µg/mL) (Figure 2C). These results indicated that *P. juliflora* extracts exhibit selectivity in reducing the viability of cancerous but not normal cells (Table 2), where the selective index of *P. juliflora* towards A549 and SK-OV-3 cells was 3.03 and 2.57, respectively (Table 2). These findings indicate that *P. juliflora* exhibited the strongest in vitro cytotoxic activity among the tested *Prosopis* extracts, with preferential cytotoxicity toward A549 and SK-OV-3 cells relative to HDFn fibroblasts.

The Alamar Blue viability assay primarily measures cellular metabolic activity, thereby providing an indirect assessment of cell viability. Direct microscopic examination, which provides direct observation of changes in cell number, integrity, and morphology, substantiated the findings of the Alamar Blue assay (Figure 3). At 50 µg/mL, *P. cineraria* and *P. farcta* extracts caused a minimal decrease in the cell numbers in all three cell lines. In contrast, *P. juliflora* caused an apparent decrease in the number of A549 and SK-OV-3 cells, with normal HDFn cells being largely unaffected (Figure 3), supporting the selective anticancer activity of the *P. juliflora* extract.

To further confirm the cell viability findings using assays that directly measure cell viability, crystal violet (Figure 4) and DAPI (Figure 5) staining assays were employed. The three cell lines exposed to varying concentrations (0, 12.5, 25, and 50 μg/mL) of extracts from *P. cineraria*, *P. farcta*, and *P. juliflora* underwent staining with the nuclear dye DAPI (Figure 5) and crystal violet (Figure 4), which stains adherent cells. Both staining techniques confirmed the cell viability results, *P. cineraria* and *P. farcta* extracts had minimal effects on the 3 cell lines at the tested concentrations (Figure 4 and Figure 5). In contrast, *P. juliflora* induced marked cytotoxicity and morphological changes in A549 and SK-OV-3 cells, with minimal impact on normal HDFn cells (Figure 4 and Figure 5), confirming its selective anticancer activity.

### 2.3. P. juliflora Leaf Extract Reduces the Protein Levels of Proliferation and Survival Markers in Human Ovarian and Lung Cancer Cells

To mechanistically and molecularly confirm the effect of the *P. juliflora* on cell viability of A549 and SK-OV-3 cells, Western blot analysis was conducted investigating the expression of key proliferation and cell cycle regulatory proteins, Ki67, AKT, P38, and Cyclin D1 (Figure 6 and Figure 7). The proliferation marker Ki67 was significantly suppressed in 50 µg/mL *P. juliflora*-treated but not *P. cineraria*-treated A549 and SK-OV-3 cells (Figure 6). Similarly, treatment with *P. juliflora* extracts led to a significant downregulation of AKT and Cyclin D1 in both cell lines compared to untreated controls. *P. cineraria* extract had no significant effect on these markers (Figure 6); AKT is a key protein involved in cell survival and migration pathways, and cyclin D1 is a critical regulator of the G1/S cell cycle transition.

These results support the inhibitory effects of *P. juliflora* on the growth of lung and ovarian cancer cells, consistent with attenuation of cell proliferation and survival signaling pathways.

Moreover, the potential involvement of stress-related signaling in the antiproliferative effects of *P. juliflora* and *P. cineraria* was assessed by examining the activation of the P38 MAPK signaling pathway in A549 and SK-OV-3 cells using Western blotting (Figure 7).

The results revealed distinct, cell-type- and extract-specific responses. In A549 cells, *P. cineraria* significantly increased the levels of phosphorylated P38 (p-P38), while *P. juliflora* significantly suppressed p-P38 expression (Figure 7A). In contrast, in SK-OV-3 cells, p-P38 levels were significantly enhanced by *P. juliflora*, but significantly decreased by *P. cineraria* treatment (Figure 7B). Total p38 protein levels remained unchanged across treatments in both cell lines (Figure 7).

These findings highlight the distinct regulatory effects of the two extracts on the p38 MAPK pathway in lung versus ovarian cancer cells, which may contribute to their context-dependent anticancer activities. These dual effects of the extracts on p38 activation between lung and ovarian cancer cells suggest a context-dependent modulation of stress-activated signaling pathways. The ability of *P. juliflora* to activate p38 in SK-OV-3 cells, but suppress it in A549 cells, may reflect differential roles of the p38 pathway in cell survival or apoptosis depending on context [45,46,47,48], and support the notion that *P. juliflora* may engage distinct molecular mechanisms to mediate its selective cytotoxic effects.

### 2.4. P. juliflora Leaf Extract Induces Intrinsic Apoptosis Pathway in Human Ovarian and Lung Cancer Cells

To investigate whether the cytotoxic effects of *P. juliflora* are mediated through the induction of apoptosis, we assessed the expression of key apoptosis-related proteins (BAX, Bcl-2, and Caspase-3), via Western blotting, in A549 and SK-OV-3 cells, following treatment with *P. juliflora* and *P. cineraria* (Figure 8).

In A549 cells, both extracts significantly upregulated the pro-apoptotic protein BAX, with *P. juliflora* producing a markedly stronger effect (Figure 8A,C). Conversely, Bcl-2, an anti-apoptotic marker protein, was downregulated by both extracts, but the reduction was statistically significant only with *P. juliflora* treatment (Figure 8A,C). Consequently, the BAX/Bcl-2 ratio, a key indicator of the activation of the mitochondrial-dependent apoptotic pathway, was increased by both extracts (Figure 8B). However, the increase in ratio was only significant in the case of *P. juliflora* treatment, which caused a substantial (~10 fold) increase compared to the control (Figure 8B), indicating a substantial shift towards apoptosis.

A similar trend was observed in SK-OV-3 cells (Figure 8D,E). *P. juliflora* significantly raised BAX expression while suppressing Bcl-2 levels, leading to a significant ~10-fold elevation in the BAX/Bcl-2 ratio (Figure 8D,E). *P. cineraria* significantly elevated BAX levels without affecting Bcl-2 levels, and slightly, but not significantly, raised the BAX/Bcl-2 ratio in SK-OV-3 cells (Figure 8D,E).

Additionally, *P. juliflora* significantly reduced the levels of the inactive form of the apoptosis executioner enzyme Caspase-3 (pro-Caspase-3) and robustly increased the levels of its activated, cleaved form (c-Caspase-3) in both cell lines (Figure 8A,C,D,F), confirming activation of the apoptotic cascade. *P. cineraria* caused a non-significant increase in pro-Caspase-3 and a significant, modest increase in c-Caspase-3 levels in A549 cells. *P. cineraria* induced a significant decrease in the levels of both pro- and c-Caspase-3 (Figure 8D,F).

These results confirm that *P. juliflora* triggers apoptosis through the intrinsic (mitochondrial) pathway involving BAX upregulation, Bcl-2 suppression, and Caspase-3 activation.

### 2.5. Prosopis Species from Qatar Leaf Extracts Inhibit the Migration of Human Ovarian and Lung Cancer Cells

To further evaluate the ability of the three extracts at attenuating the metastatic phenotype of cancer cells, we evaluated their effect on cell migration, a hallmark of cancer cell metastasis, using a scratch/wound healing assay (Figure 9).

A scratch was induced through a confluent cell monolayer of A549 and SK-OV-3 cells and subsequently treated with 50 µg/mL of each extract for 48 h. Untreated control A549 cells exhibited extensive wound closure, consistent with active cell migration (Figure 9A,B). Treatment with *P. cineraria* did not significantly affect cell migration, whereas *P. farcta* induced a mild but statistically significant reduction in scratch closure rate (*p* < 0.05) (Figure 9A,B). Notably, *P. juliflora* treatment led to a pronounced and significant inhibition of scratch closure (*p* < 0.001) (Figure 9A,B), leaving the scratch area largely unhealed and indicating potent anti-migratory activity.

For the SK-OV-3 cells, control groups demonstrated rapid and near-complete wound closure within 48 h (Figure 9C,D). In contrast, all three plant extracts significantly impaired migration, with *P. juliflora* exhibiting the most potent anti-migratory effect (*p* < 0.001) (Figure 9C,D). The scratch areas in treated cells remained substantially open, confirming reduced cell motility.

These results suggest that all the *Prosopis* species from Qatar could attenuate cancer cell migration in both lung and ovarian cancer cells. *P. juliflora* showed the most potent anti-migratory effects, which may contribute to its broader anticancer potential by interfering with metastatic processes.

## 3. Discussion

Our current study investigated the anticancer potential of *P. cineraria*, *P. farcta*, and *P. juliflora* extracts on lung (A549) and ovarian (SK-OV-3) cancer cell lines, alongside their effects on normal human dermal fibroblast neonatal (HDFn) cells. A combination of assays including GC–MS analysis and a range of cellular and molecular assays were employed to investigate the chemical composition, cytotoxicity, migration inhibition, and alterations in protein expression related to cell proliferation and apoptosis, providing a preliminary mechanistic insight into their anticancer therapeutic potential.

The GC–MS profiling revealed 36 low-molecular-weight phytochemicals including terpenes, terpenoids, sterols, alkanes, alcohols, ketones, and epoxides, with several compounds shared across the three species. Notably, aromadendrene, caryophyllene oxide, nootkatone, and lupeol, which were detected in all plants, have been demonstrated to have anticancer properties with diverse mechanisms of action. Aromadendrene derivatives usually induce mitochondrial apoptosis through ROS generation and caspase activation [49]. Caryophyllene oxide suppresses proliferation via MAPK activation and inhibition of PI3K/AKT/mTOR and STAT3 pathways [50,51]. Nootkatone functions as an AMPK activator and inhibits tumor growth by promoting oxidative stress, G2/M arrest, and ATF4-CHOP pathway activation [52,53]. Lupeol exerts antiproliferative effects through inhibition of Wnt/β-catenin signalling and modulation of Ras-dependent downstream cascades, including PKCα, PI3K/AKT, MAPKs, and NF-κB [54,55]. The presence of these compounds across the extracts may contribute to the biological activities observed in A549 and SK-OV-3 cells.

Several compounds were detected only in *P. juliflora* under the present GC–MS conditions, including α-curcumene, humulene, and (E)-limonene oxide, and may represent candidate contributors to the stronger biological activity observed with this extract. Curcumene-related compounds have been shown to act as antiproliferative, pro-apoptotic, and cell-cycle-arrest mediators across multiple cancer types and have advanced to clinical evaluation [56,57,58,59,60,61]. Humulene have demonstrated selective toxicity toward cancer cells, suppressing proliferation and inducing apoptosis in breast, liver, pancreatic, and colorectal cancers while sparing normal fibroblasts [62,63,64,65,66]. Limonene derivatives have inhibited tumor growth by inducing G2/M arrest, promoting mitochondrial apoptosis, and reducing cyclin D1; importantly, limonene has reached clinical trials in breast cancer, demonstrating safety and pharmacodynamic activity [67,68,69]. Overall, the unique compounds detected in *P. juliflora* may represent candidate contributors to the stronger biological activity observed with this extract.

Although GC–MS is known to underrepresent polar and high-molecular-weight phytochemicals such as phenolic acids, flavonoids, and glycosides, it was used for preliminary phytochemical screening in the used extracts. More advanced HPLC–UV/PDA or LC–MS/MS platforms are planned to be used in the future for accurate extracts profiling [70]. This GC–MS profiling was not designed to provide a full structural profile, but rather to deliver structural characterization of low-molecular-weight, GC-amenable metabolites, in accordance with best-practice recommendations for ethnopharmacological and natural-products research, including the Four Pillars of Best Practice in Ethnopharmacology and the ConPhyMP guidelines [71]. Accordingly, the present analysis serves as a chemically informative validation of extract composition that supports the biological assays [72], while future studies will incorporate HPLC–MS–based profiling to capture a broader spectrum of polar metabolites that are not detectable by GC–MS.

The cell viability assays demonstrated that *P. cineraria* and *P. farcta* exerted minimal or only mild cytotoxic effects at the tested concentrations (12.5–50 µg/mL), with slight reductions observed primarily in SK-OV-3 ovarian cancer cells, while largely sparing A549 lung cancer cells and non-cancerous HDFn fibroblasts. The relatively higher sensitivity of SK-OV-3 cells may reflect the increased susceptibility of ovarian cancer cells to oxidative stress and phytochemical-induced metabolic disruption [15,73,74]. These findings are consistent with our GC–MS analysis, which identified caryophyllene oxide, nootkatone, and lupeol in both extracts—compounds previously associated with ROS-mediated apoptosis and stress-related cytotoxicity in cancer cells [73]. The mild activity observed in the present study is also in agreement with the limited available literature, where anticancer effects of *P. farcta* and *P. cineraria* were generally reported at substantially higher concentrations. For example, *P. farcta* exhibited cytotoxic activity against A549 and HT-29 cells at concentrations ranging from 100 to 800 µg/mL [75], while *P. cineraria* demonstrated selective cytotoxicity toward MCF-7 breast cancer cells with minimal effects on normal fibroblasts [23]. Together, these findings suggest that both species possess biologically relevant anticancer properties, although their activity within the present experimental range appears weaker than that of *P. juliflora*. Future studies using higher concentrations and bioactivity-guided fractionation are therefore needed to better define the therapeutic potential and active constituents of *P. cineraria* and *P. farcta*.

In contrast, *P. juliflora* exhibited dose-dependent cytotoxicity toward A549 and SK-OV-3 cells, with IC50 values of 42.2 µg/mL and 49.8 µg/mL, respectively. Importantly, the *P. juliflora* IC50 in normal HDFn cells (128 µg/mL) yielded selectivity index (SI) values of 3.03 for A549 and 2.57 for SK-OV-3, indicating preferential cytotoxicity toward the tested cancer cells. These initial in vitro selectivity values support further investigation of *P. juliflora* as a source of candidate anticancer constituents, while not establishing a broad therapeutic window for the crude extract. These findings align with previous studies demonstrating that *P. juliflora* extracts and alkaloid fractions exhibit antiproliferative and selective cytotoxic activity against several human cancer cell lines, including MCF-7, MDA-MB-231, HepG2, and LS-174T, while sparing normal keratinocytes or fibroblasts [15,18,76].

The stronger biological activity observed with *P. juliflora* coincided with the detection of several bioactive constituents, including caryophyllene oxide, lupeol, nootkatone, α-curcumene, humulene, and (E)-limonene oxide. These compounds have well-documented anticancer activities, including induction of apoptosis, suppression of PI3K/AKT/mTOR signaling, inhibition of STAT3 activation, cell-cycle arrest, modulation of ROS levels, and autophagy-driven cell death [52,77,78,79,80,81,82,83,84]. The reported biological activities of these constituents support their consideration as potential contributors to the effects observed with *P. juliflora*, although their individual or combined contributions cannot be established from the present study.

Microscopic examination, crystal violet staining, and DAPI nuclear staining supported the Alamar Blue findings. *Prosopis cineraria* and *P. farcta* extracts induced only minor morphological changes in A549 and SK-OV-3 cells and had no detectable effect on HDFn cells, confirming their low cytotoxicity at the tested doses. In contrast, *P. juliflora* extract induced pronounced morphological hallmarks of apoptosis—cell shrinkage, detachment, chromatin condensation, and nuclear fragmentation—specifically in cancer cells, with minimal effects on HDFn cells. Similar selective apoptotic activity has been reported in *P. juliflora*-treated breast cancer models, where it selectively targeted metastatic MDA-MB-231 cells but not normal HaCaT cells [18]. The nuclear condensation patterns observed here are consistent with apoptosis rather than necrosis, further supported by dose-dependent reductions in viable DAPI-positive nuclei [32]. Collectively, these findings identify *P. juliflora* extract as the most biologically active *Prosopis* species evaluated in this study.

Western blot analysis provided clear mechanistic insight into the anticancer actions of *P. juliflora* and *P. cineraria* extracts in A549 and SK-OV-3 cells. Treatment with *P. juliflora* extract markedly increased the expression of the pro-apoptotic proteins BAX and cleaved caspase-3 while reducing Bcl-2 and pro-caspase-3 levels, consistent with activation of the intrinsic mitochondrial apoptotic pathway. The resulting large increase in the BAX/Bcl-2 ratio, particularly in *P. juliflora*-treated cells, indicates mitochondrial outer membrane permeabilization and downstream caspase activation. These findings align with prior studies showing that *P. juliflora* induces BAX upregulation and caspase-3 activation in melanoma and breast cancer models [18,34]. This apoptotic activity of the extract might be linked to the bioactive constituents identified, including α-curcumene, humulene, and (E)-limonene oxide, all of which have been reported to cause pro-apoptotic or oxidative-stress–mediated cytotoxic effects. Curcumene-related compounds, for example, can induce apoptosis in lung and ovarian cancer cells by suppressing Bcl-2 and pro-caspase-3 and enhancing BAX and cleaved caspase-3 [85,86,87,88]. Humulene has similarly been demonstrated to promote apoptosis in hepatocellular carcinoma by lowering pro-caspase-3 and increasing cleaved caspase-3 [66], while limonene has been reported to trigger apoptosis through BAX upregulation and Bcl-2 suppression in lung and bladder cancer [84,89].

Consistent with the cell viability findings, *P. juliflora* extract reduced the levels of the proliferation- and survival-associated proteins Ki67, AKT, and Cyclin D1 more effectively than *P. cineraria* extract. These effects mirror the known anti-proliferative mechanisms of curcumin, which inhibits the PI3K/AKT pathway and downregulates Cyclin D1 in lung, ovarian, and gastric cancers [90,91,92,93]. Humulene likewise is known to inhibit Cyclin D1 expression in hepatocellular carcinoma, reinforcing its relevance as a proliferation-modulating constituent [94]. However, because phosphorylated AKT was not assessed, the observed reduction in total AKT alone does not establish inhibition of AKT pathway activation, warranting future assessment of phosphorylated AKT levels relative to total AKT.

The two extracts exhibited opposite effects on the p38 MAPK pathway, highlighting the context-dependent nature of stress-activated signaling in cancer cells. In A549 cells, *P. cineraria* increased p38 phosphorylation, whereas *P. juliflora* suppressed it. In contrast, in SK-OV-3 cells, *P. juliflora* enhanced p38 activation, while *P. cineraria* reduced it. These divergent responses align with the dual role of p38, which can either promote apoptosis or support cell survival depending on the cellular context, upstream stimulus, and tumor type [45,46,47,48]. The ability of *P. juliflora* to activate p38 in SK-OV-3 cells but suppress it in A549 cells suggests that this extract may engage distinct molecular programs across cancer types. This interpretation is supported by previous studies showing that plant-derived extracts can either inhibit p38 signaling [95,96] or activate it [97,98,99], further emphasizing that complex phytochemical mixtures may regulate stress pathways in a cell-type- and extract-dependent manner. These findings suggest that p38 activation in SK-OV-3 cells may contribute to the pro-apoptotic effects of *P. juliflora* extract, whereas suppression of p38 in A549 cells may reflect inhibition of stress-adaptive survival signaling.

*Prosopis juliflora* extract produced the greatest reduction in wound closure among the three extracts in both A549 and SK-OV-3 cells, supporting its potential to interfere with cancer-cell migratory behavior and, consequently, suggesting possible anti-metastatic activity. *Prosopis farcta* produced moderate inhibition of migration in A549 cells and a more marked suppression in SK-OV-3 cells, indicating a greater effect on ovarian cancer cell motility. In contrast, *P. cineraria* exerted little effect on A549 migration but significantly impaired SK-OV-3 movement, consistent with its selective activity toward ovarian cancer. These findings align with previous reports describing anti-migratory and potential anti-metastatic effects of *P. juliflora*, including reduced cancer-cell adhesion and migration [18,34], and support the broader potential of *Prosopis* species as sources of natural inhibitors of cancer cell migration.

Several limitations should be considered when interpreting the findings of the present study. All experiments were conducted under in vitro conditions using one lung and one ovarian cancer model and a single non-malignant comparator. These models cannot capture the molecular and histopathological heterogeneity of lung and ovarian cancers, and the present findings should therefore not be generalized across their diverse molecular subtypes. Although the selectivity indices indicated preferential cytotoxicity toward A549 and SK-OV-3 cells relative to HDFn fibroblasts, these initial in vitro findings do not establish a broader therapeutic window or safety profile. Validation across additional malignant and non-malignant cell models, followed by in vivo studies, is therefore warranted to further evaluate efficacy, selectivity, and biological relevance. The use of crude extracts precludes definitive attribution of the observed anticancer activity of *P. juliflora* to individual phytochemicals, particularly because multiple herbal constituents may act additively or synergistically to produce the overall biological response. In addition, GC–MS primarily detects low-molecular-weight, GC-amenable constituents and may underrepresent polar and higher-molecular-weight compounds such as phenolic acids, flavonoids, and glycosides. Because the present GC–MS analysis was qualitative rather than quantitative, direct correlations between constituent abundance and biological activity cannot be established. Future studies combining bioactivity-guided fractionation with complementary analytical platforms such as HPLC or LC-MS/MS should therefore evaluate both isolated constituents and defined combinations, including α-curcumene, humulene, and (E)-limonene oxide, to clarify their individual contributions and potential synergistic effects. Mechanistic interpretation is also limited by the use of selected protein-expression markers. In particular, apoptosis was assessed primarily by Western blot analysis of BAX, Bcl-2, and caspase-3; complementary quantitative approaches, including flow-cytometric apoptosis assays and cell-cycle analysis, will be required for more comprehensive characterization. Similarly, only total AKT expression was assessed, without measuring phosphorylated AKT relative to total AKT, thereby limiting conclusions regarding pathway activation.

## 4. Materials and Methods

### 4.1. Collection of Plant Material and Preparation of Plant Extracts

Leaf extracts of *P. farcta*, *P. cineraria*, and *P. juliflora* were prepared as we have previously described [29]. Our previous optimization studies evaluated different extraction conditions [29], and the established 70% ethanol protocol was retained for the present study. Briefly, leaves were collected from designated sites in Qatar, washed, oven-dried at 45 °C for 5 days to ensure total dryness, and ground into a fine powder [29]. The powdered material (20 g) was extracted with 70% ethanol (200 mL) at 45 °C for 48 h under shaking. Extracts were centrifuged, allowed to dry, and the resulting residues were scraped, collected, and stored at 4 °C. The extraction yield was approximately 10–11% (*w*/*w*), corresponding to approximately 2 g of crude extract from 20 g of dried leaf material. The same extraction conditions were applied to all preparations, including the plant material-to-solvent ratio, ethanol concentration, extraction temperature, duration, and shaking conditions. Fresh young leaves were consistently selected before drying and extraction to minimize batch-to-batch variability.

Stock solutions (100 mg/mL) were prepared in sterile double-distilled water and further sterilized by a syringe filter. The stock solutions were subsequently diluted to 10 mg/mL in serum-free culture medium and further diluted in culture medium to the concentrations used for experiments, including cell treatment. No separate vehicle control was included; cells maintained in culture medium without extract served as untreated negative controls. Because the aqueous stock solutions were serially diluted before treatment, the maximum amount of double-distilled water introduced into the final treatment medium was approximately 0.075% (*v*/*v*) at the highest tested concentration of 75 µg/mL, representing only a minimal change in the final medium composition. The ethanol used for extraction was removed during preparation of the dried crude extracts and was therefore not intentionally introduced into the cell-treatment medium; accordingly, no separate water or ethanol vehicle control was included.

### 4.2. Gas Chromatography/Mass Spectrometry (GC–MS) Identification of Prosopis Leaf Extracts

The low molecular weight compounds of *P. cineraria*, *P. farcta*, and *P. juliflora* leaf extracts were analyzed using GC–MS via a SHIMADZU GC/MS-TQ 8040 NX system (Shimadzu, Tokyo, Japan) with a Restek RTxi-5 Sil MS (30 m × 0.25 mm ID × 0.25 µm) GC capillary column (Restek, Bellefonte, PA, USA), as previously described [2,100]. The injection volume was one μL in split ratio mode (1:10). The injector inlet and transfer line temperatures were held at 300 °C and 200 °C, respectively. The column oven temperature was initially set to 80 °C (held for 1 min), then ramped to 280 °C at 5 °C/min (held for 10 min). Helium gas was used as the carrier gas at a flow rate of 1.5 mL/min and a pressure of 23.1 KPa. The mass spectrometer was run in electron ionization (EI) mode at an ion source temperature of 250 °C, an ionization energy of 70 eV, and a scan range of *m*/*z* 50–600. The *Prosopis* compounds were identified by comparing their retention times and mass spectra with those of reference compounds stored in the National Institute of Standards and Technology (NIST version 20) database.

### 4.3. Cell Culture

Human lung adenocarcinoma A549 [American Type Culture Collection (ATCC, Manassas, VA, USA) CCL-185], ovarian adenocarcinoma SK-OV-3 (ATCC HTB-77), and normal neonatal fibroblast (HDFn; ATCC PCS-201-010) were procured from ATCC. All cell lines were maintained in a humidified incubator at 5% CO_2_ and 37 °C. A549 cells were grown in RPMI-1640 medium supplemented with 2.0 mM L-glutamine, 1% penicillin/streptomycin (100 U/mL and 100 µg/mL, respectively), and 10% heat-inactivated fetal bovine serum (FBS; qualified Brazil Gibco™; Thermo Fisher Scientific, Waltham, MA, USA). SK-OV-3 and HDFn cells were cultured in DMEM medium with the same supplements. Cells were passaged using Trypsin-EDTA (ref 3920459; VWR, Lutterworth, Leicestershire, UK), and most experiments were conducted when the cells reached 70–80% confluence.

### 4.4. Assessment of Cell Viability

#### 4.4.1. Alamar Blue Cell Viability Assay

The alamarBlue™ cell viability reagent (Invitrogen™, Thermo Fisher Scientific) was used to assess the effects of the *Prosopis* extracts across two established human cancer models and a non-malignant comparator: A549, SK-OV-3, and HDFn cells. Human lung adenocarcinoma A549 cells (ATCC CCL-185), ovarian adenocarcinoma SK-OV-3 cells (ATCC HTB-77), and HDFn fibroblasts (ATCC PCS-201-010) were obtained from the American Type Culture Collection (ATCC, Manassas, VA, USA). A549 and SK-OV-3 cells were selected as human lung and ovarian adenocarcinoma models, respectively, consistent with the established use of human tumor cell lines in preclinical anticancer drug screening [36]. HDFn cells were included as a primary, non-malignant comparator to assess differential cytotoxicity and calculate the in vitro selectivity index of the extracts, an approach used to distinguish preferential anticancer activity from general cytotoxicity in natural-product bioassays [37]. A549, SK-OV-3, and HDFn cells were seeded in 96-well tissue culture plates at a density of 4.0 × 10^3^, 5.0 × 10^3^, or 1.0 × 10^4^ cells per well, respectively, and incubated for 24 h. The cells were then treated with 12.5, 25, 50, or 75 µg/mL of each extract or left untreated as a control. Cellular viability was assessed 48 h post-treatment by washing the cells with 1X PBS and adding 100 µL of cell culture medium containing 10% (*v*/*v*) alamarBlue™ reagent. The cells were incubated for an additional 4 h and their fluorescence was measured at 560 nm (excitation) and 590 nm (emission) using a TECAN Infinite M200 plate reader (Männedorf, Switzerland). Cell viability was expressed with respect to control untreated cells, the viability of which was set as 100% viability, according to the following formula: % cell viability = (fluorescence of treated cells/fluorescence of untreated control cells) × 100. Each treatment was tested in triplicate wells, and each experiment was independently repeated three times (*n* = 3).

#### 4.4.2. Microscopic Examination

Cells were seeded in 96-well tissue culture plates as above and treated 24 h later with 50 µg/mL of each of the three extracts. Untreated cells served as negative controls. At 48 h following treatment, cellular morphology was observed and photographed using an inverted microscope (EVOS^®^ FL Cell Imaging System, Invitrogen™, Thermo Fisher Scientific).

#### 4.4.3. Crystal Violet Staining

Crystal violet (HiMedia Laboratories, Maharashtra, India) staining was employed to confirm the effects of the plant extracts on cell morphology and viability. A549 and SK-OV-3 were seeded at a density of 2.5 × 10^4^ cells per well in 24-well cell culture plates for 24 h. The cells were then treated with 12.5, 25, or 50 µg/mL of the plant extracts, or left untreated as a control. After 48 h of treatment, the cells were washed with 1X PBS, fixed using PBS-buffered 4.0% formaldehyde for 15 min, and subsequently stained with 0.5% crystal violet (*v*/*v* in 80% methanol; HiMedia Laboratories, Maharashtra, India) for an additional 15 min. The cells were then washed with 1X PBS to remove excess dye. Cellular morphology and viability were subsequently examined and images taken using Invitrogen™ EVOS^®^ FL Cell Imaging System.

#### 4.4.4. DAPI Nuclear Staining

A549 and SK-OV-3 cells were seeded and treated as described in the Alamar Blue assay methods with 12.5, 25, or 50 µg/mL of each extract, and untreated cells were used as a control. Following 48 h of treatment, the cells were washed with 1X PBS and 4% PBS-buffered formaldehyde was used to fix the cells for 15 min. The cells were washed with 1X PBS and stained for 10 min in the dark with 100 µL of 1 µg/mL DAPI solution (Thermo Scientific/Invitrogen, cat# D1306), as a nuclear stain. Finally, fluorescent cells were observed, imaged, and quantified using BioTek Cytation 5 reader (BioTek; Agilent Technologies, Winooski, VT, USA).

### 4.5. Scratch/Wound Healing Assay

A scratch/wound healing assay was used to examine the impact of the plant extracts on the migratory properties of the metastatic cell lines: SK-OV-3 and A549 cells. Cells were seeded in a 6-well cell culture plate and allowed to grow to a confluent cell monolayer. A scratch was introduced into the monolayer using a sterile 1000 µL blue micropipette tip. The cells were washed with 1X PBS to remove cell debris and then treated for 48 h with 50 µg/mL of the plant extracts or left untreated as a control. Images were captured under an inverted light microscope (EVOS^®^ FL Cell Imaging System) at 0 and 48 h to record the average width of the introduced scratch at a minimum of five random points along its length. NIH ImageJ software version 1.54g (https://imagej.net/ij/, accessed on 30 April 2026) was used to analyze the images and measure the average wound closure between 0 and 48 h using the following formula:

Wound closure = (average width at 0 h − average width at 48 h)/average width at 0 h.

### 4.6. Western Blotting Analysis

A549 and SK-OV-3 cells were cultured in T75 flasks and allowed to reach 70–80% confluence when they were treated with 50 µg/mL of the plant extracts or left untreated as a control. After 48 h of treatment, the cells were washed with 1X PBS, lysed using a 1X cell lysis buffer [ref 9803S, Cell Signaling Technology, Inc. (CST), Danvers, MA, USA] containing 1X Roche Complete protease inhibitor (ref 11697498001; Roche, Sigma-Aldrich, St. Louis, MO, USA) for 5 min on ice, scraped, collected, and subjected to centrifugation at 1.4 × 10^4^ rpm for 15 min at 4 °C. Protein concentration of the total cell lysate supernatants was quantified using Pierce™ BCA Protein Assay Kit (ref A53225; Thermo Fisher Scientific, Rockford, IL, USA). An amount of 20 μg of protein lysates was resolved using 10% SDS-PAGE and transferred to a PVDF membrane (Immobilon PVDF; Biorad, Hercules, CA, USA). The PVDF membranes were then blocked with 1X blocking buffer (ref 37520, Thermo Fisher Scientific) and incubated overnight at 4 °C with the primary antibodies: anti-rabbit Ki67 (ref 28074-1-AP; Proteintech, Rosemont, IL, USA), AKT (CST #9272s, Cell Signaling Technology), cyclin D1 (CST #55506s; Cell Signaling Technology), anti-mouse BCL2 (CST #15071S; Cell Signaling Technology), anti-rabbit BAX D2E11 (CST #5023; Cell Signaling Technology), anti-mouse P38 MAPK (ref 14064-1-AP, Proteintech), anti-rabbit phospho-p38 MAPK Thr180/Tyr182 (CST #9211, Cell Signaling Technology), anti-rabbit Caspase 3 (CST #9662, Cell Signaling Technology), anti-rabbit Caspase-3 (CST #9662; Cell Signaling Technology) for detection of full-length Caspase-3, anti-rabbit cleaved Caspase-3 (Asp175) (CST #9661; Cell Signaling Technology) for detection of cleaved Caspase-3, and anti-mouse β-actin 8H10D10 (CST #3760; Cell Signaling Technology) antibody was used to confirm equal loading of protein samples. The membranes were then washed using 1X TBST and incubated for 1.5 h with either goat anti-rabbit (CST #7074; Cell Signaling Technology) or horse anti-mouse IgG (CST #7076; Cell Signaling Technology) HRP-conjugated secondary antibodies. The membranes were washed with 1X TBST and the Immunereactive bands were detected using SuperSignal™ West Pico PLUS Chemiluminescent Substrate kit (ref 34577; Thermo Fisher Scientific) and visualized and imaged using Invitrogen™ iBright Imaging System (Thermo Fisher Scientific). Band intensities were quantified using ImageJ software. The densitometric intensity of each target protein was normalized to the corresponding β-actin band intensity, which served as the loading control. For phosphorylated p38, the p-p38 signal was normalized to the corresponding total p38 protein level. The resulting normalized values were used for statistical analysis. Western blot experiments were independently repeated three times (*n* = 3).

### 4.7. Statistical Analysis

Data analysis and graph generation were performed using GraphPad Prism 9 software (GraphPad Software Inc., San Diego, CA, USA). Results are presented as mean ± SEM. Experiments were performed in three independent biological replicates (*n* = 3), where *n* represents the number of independent experiments. Where applicable, each biological replicate included three technical replicates, representing repeated measurements within the same independent experiment (e.g., three replicate wells in the Alamar Blue assay). Statistical significance was evaluated using one-way ANOVA followed by Tukey’s post-hoc multiple-comparison test. A *p*-value < 0.05 was considered statistically significant.

## 5. Conclusions

This study provides comparative in vitro evidence of the anticancer activity of the three *Prosopis* species growing in Qatar, with *P. juliflora* showing the strongest effects against A549 lung and SK-OV-3 ovarian cancer cells under the tested conditions. GC–MS profiling revealed diverse low-molecular-weight constituents, including several compounds detected only in *P. juliflora* under the present analytical conditions, which may contribute to its stronger antiproliferative, pro-apoptotic, and anti-migratory effects. However, the individual and combined contributions of these constituents remain to be established. *P. juliflora* also reduced proliferation- and survival-associated proteins, modulated p38 MAPK signaling in a cell-type-dependent manner, and induced changes in BAX, Bcl-2, and caspase-3 consistent with activation of apoptotic pathways. In contrast, *P. cineraria* and *P. farcta* showed comparatively limited cytotoxicity at the tested concentrations but retained anti-migratory activity, particularly in SK-OV-3 cells, suggesting the presence of bioactive constituents that warrant further investigation. Overall, these findings identify *P. juliflora* as the most active of the three tested *Prosopis* extracts and support further investigation of its anticancer potential through bioactivity-guided fractionation, quantitative phytochemical profiling, additional mechanistic studies, and validation in broader in vitro and in vivo models.

## Figures and Tables

**Figure 1:**
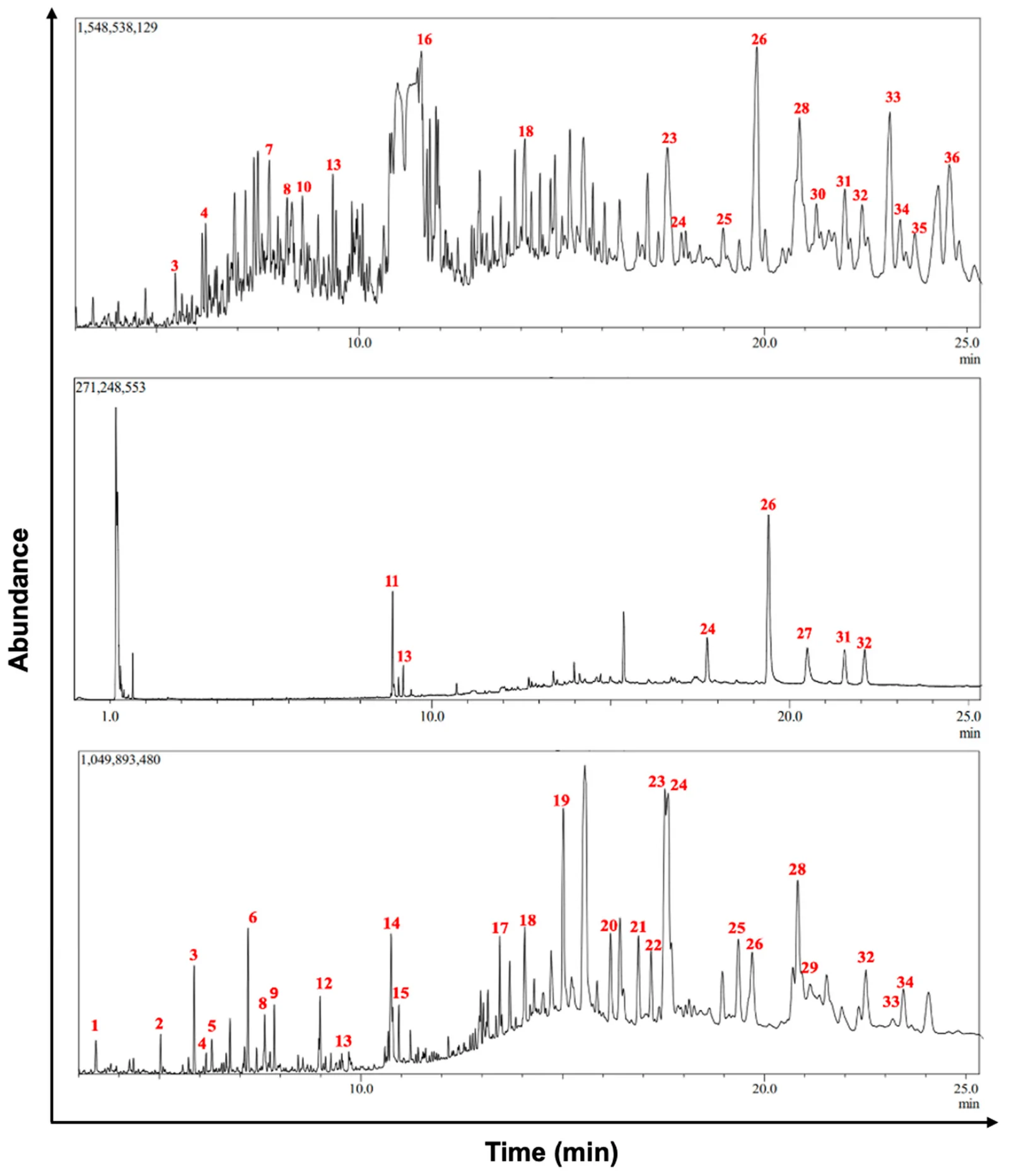
GC–MS chromatogram of *P. cineraria* (**upper panel**), *P. farcta* (**middle panel**), and *P. juliflora* (**lower panel**) leaf extracts displaying the elution of phytochemicals listed in Table 1.

**Figure 2:**
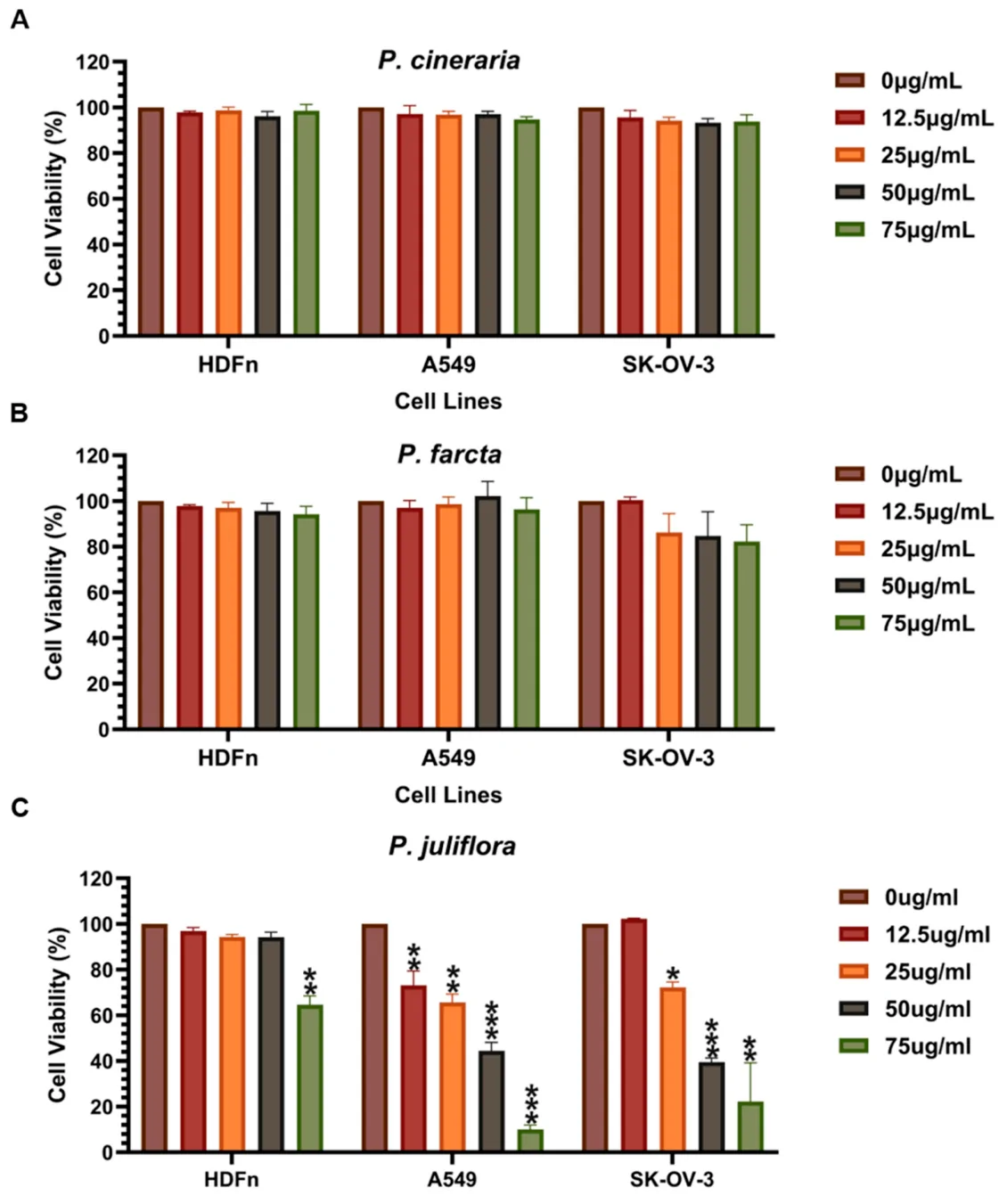
The viability of HDFn, A549, and SK-OV-3 cells upon treatment with *P. cineraria*, *P. farcta*, and *P. juliflora* leaf extracts. Cell viability was assessed 48 h following treatment with the indicated concentrations of the plant extracts using the Alamar Blue assay. (**A**) Cell viability of HDFn, A549, and SK-OV-3 cells treated with *P. cineraria* leaf extracts. (**B**) Same as in (**A**), but the cells were treated with *P. farcta* leaf extracts. (**C**) Same as in (**A**), but the cells were treated with *P. juliflora* leaf extracts. Data are presented as the mean ± SEM of three independent experiments (*n* = 3). * indicates *p* < 0.05, ** indicate *p* < 0.01, and *** indicate *p* < 0.001.

**Figure 3:**
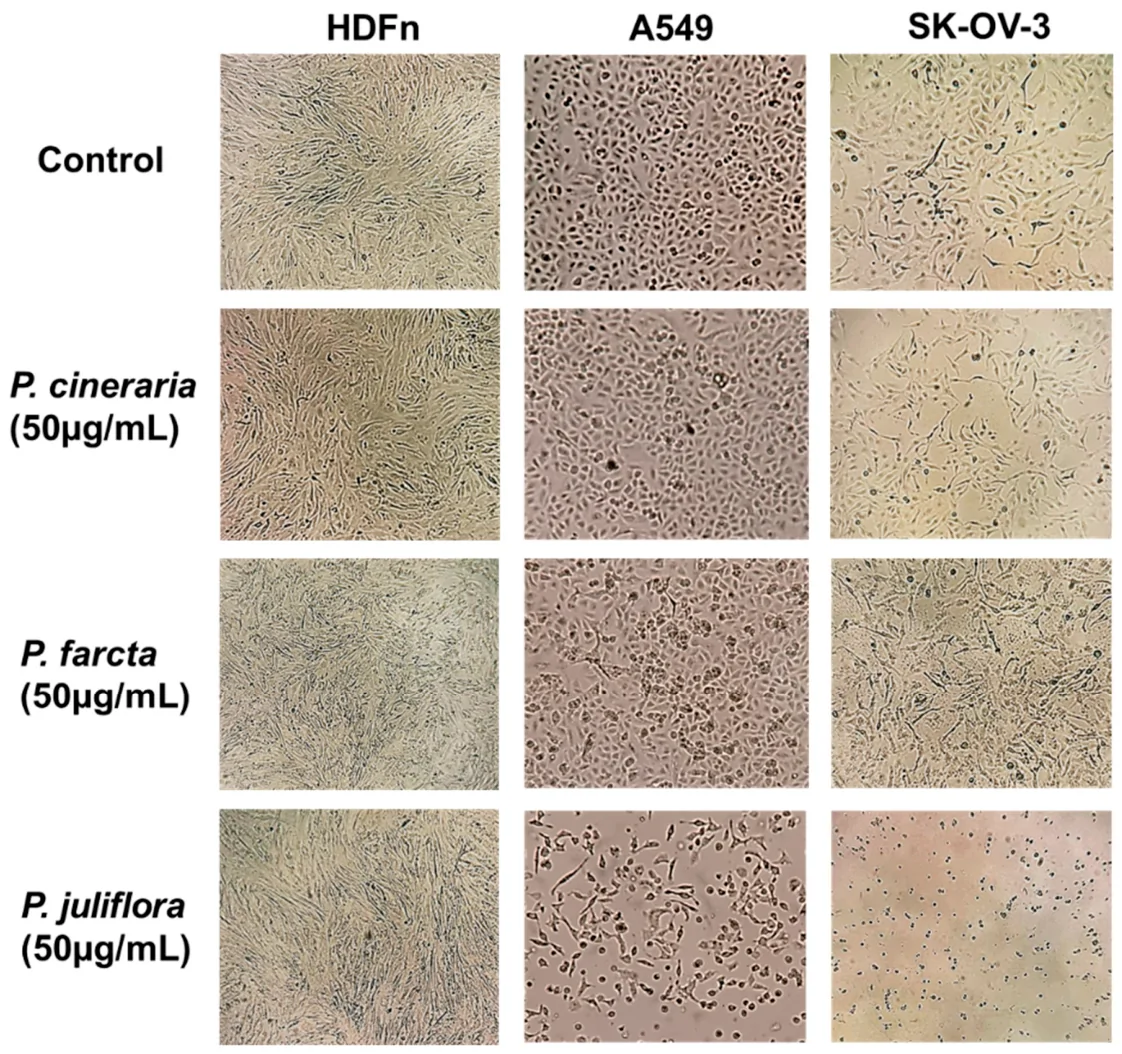
Effects of *P. cineraria*, *P. farcta*, and *P. juliflora* on viability of HDFn, A549, and SK-OV-3 cells. Cells were seeded until they reached 70–80% confluence, treated with 50 μg/mL of the extracts for 48 h and observed under phase-contrast microscopy at 10× magnification.

**Figure 4:**
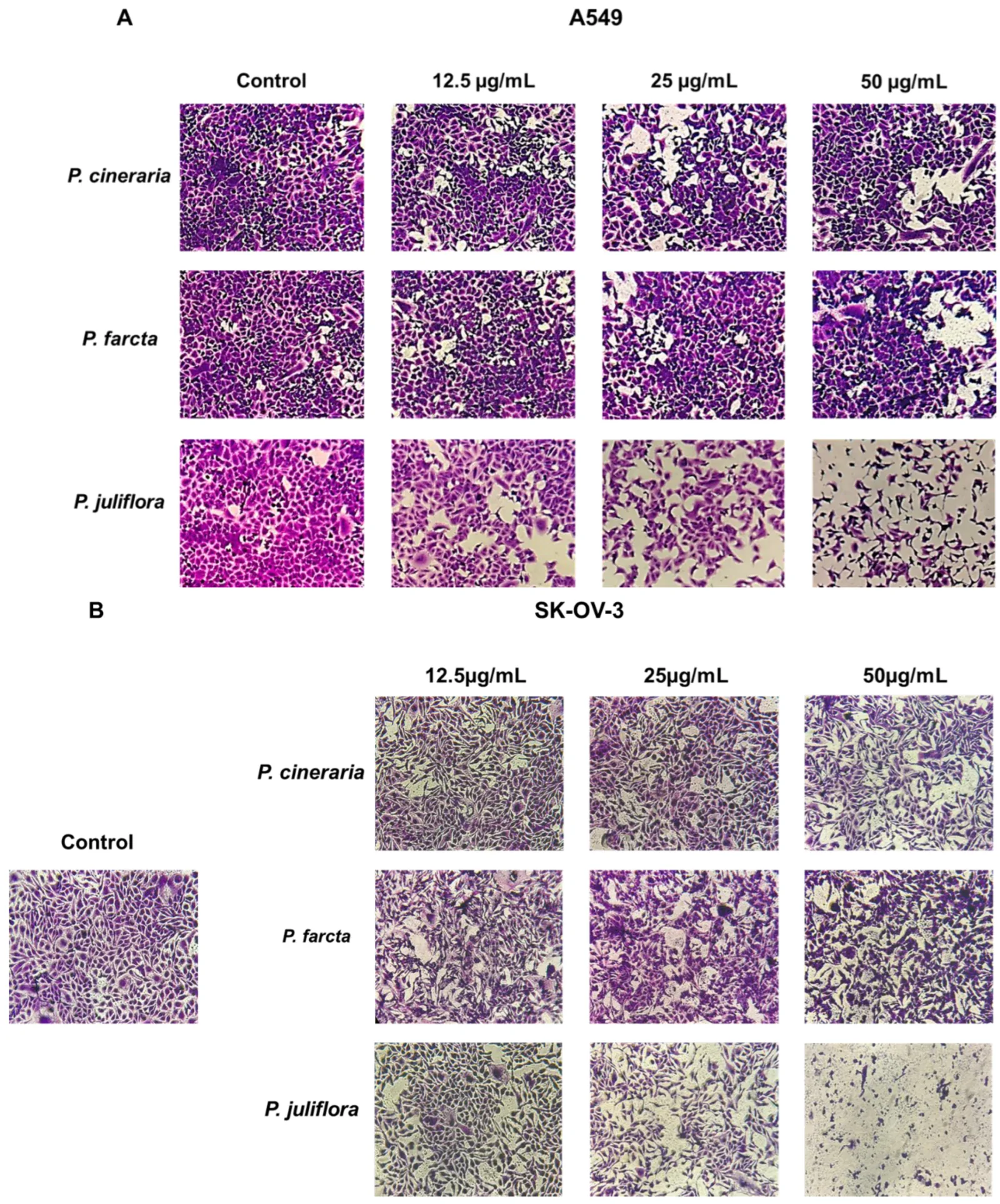
Crystal violet staining showing the effects of treatment with *P. cineraria*, *P. farcta*, and *P. juliflora* extracts on A549 and SK-OV-3 cell viability. (**A**) A549 cells were treated with the indicated concentrations of the three extracts for 48 h and stained with crystal violet. Untreated cells served as a control. (**B**) Same as in (**A**), but for SK-OV-3 cells. Both cell lines were observed under phase-contrast microscopy at 10× magnification.

**Figure 5:**
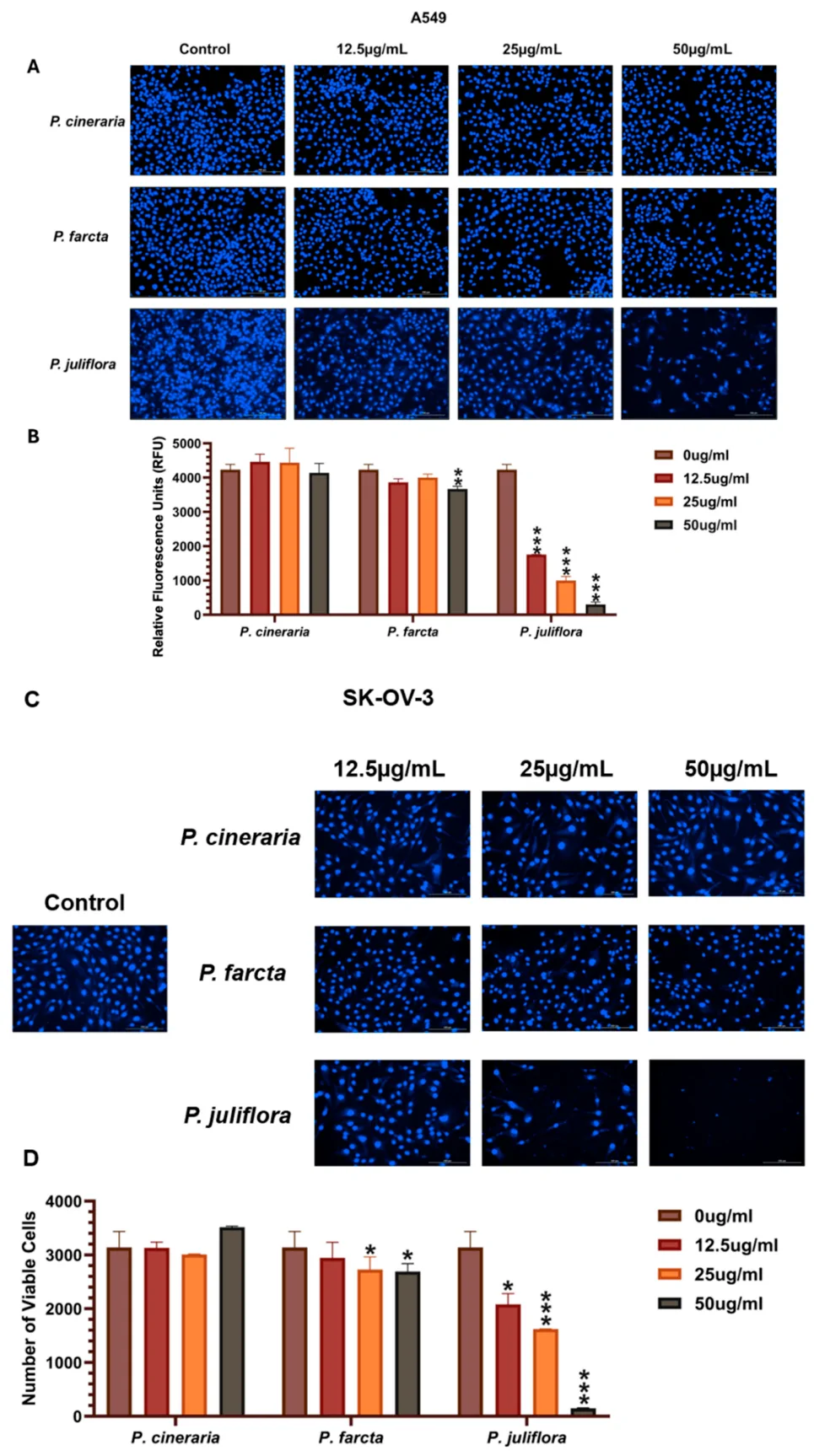
DAPI nuclear staining showing the effects of treatment with *P. cineraria*, *P. farcta*, and *P. juliflora* extracts on A549 and SK-OV-3 cell viability. (**A**) Representative DAPI-stained images of A549 cells treated with the indicated concentrations of the three extracts for 48 h. Untreated cells served as a control. (**B**) Quantification of the viable DAPI-stained nuclei in (**A**); quantification was performed using Cytation 5. (**C**) Same as in (**A**), but for SK-OV-3 cells. (**D**) Quantification of the viable DAPI-stained nuclei in (**C**), performed using Cytation 5. Data are presented as mean ± SEM of three independent experiments (*n* = 3). * indicates *p* < 0.05, ** indicate *p* < 0.01, and *** indicate *p* < 0.001. Bar scale = 200 μm.

**Figure 6:**
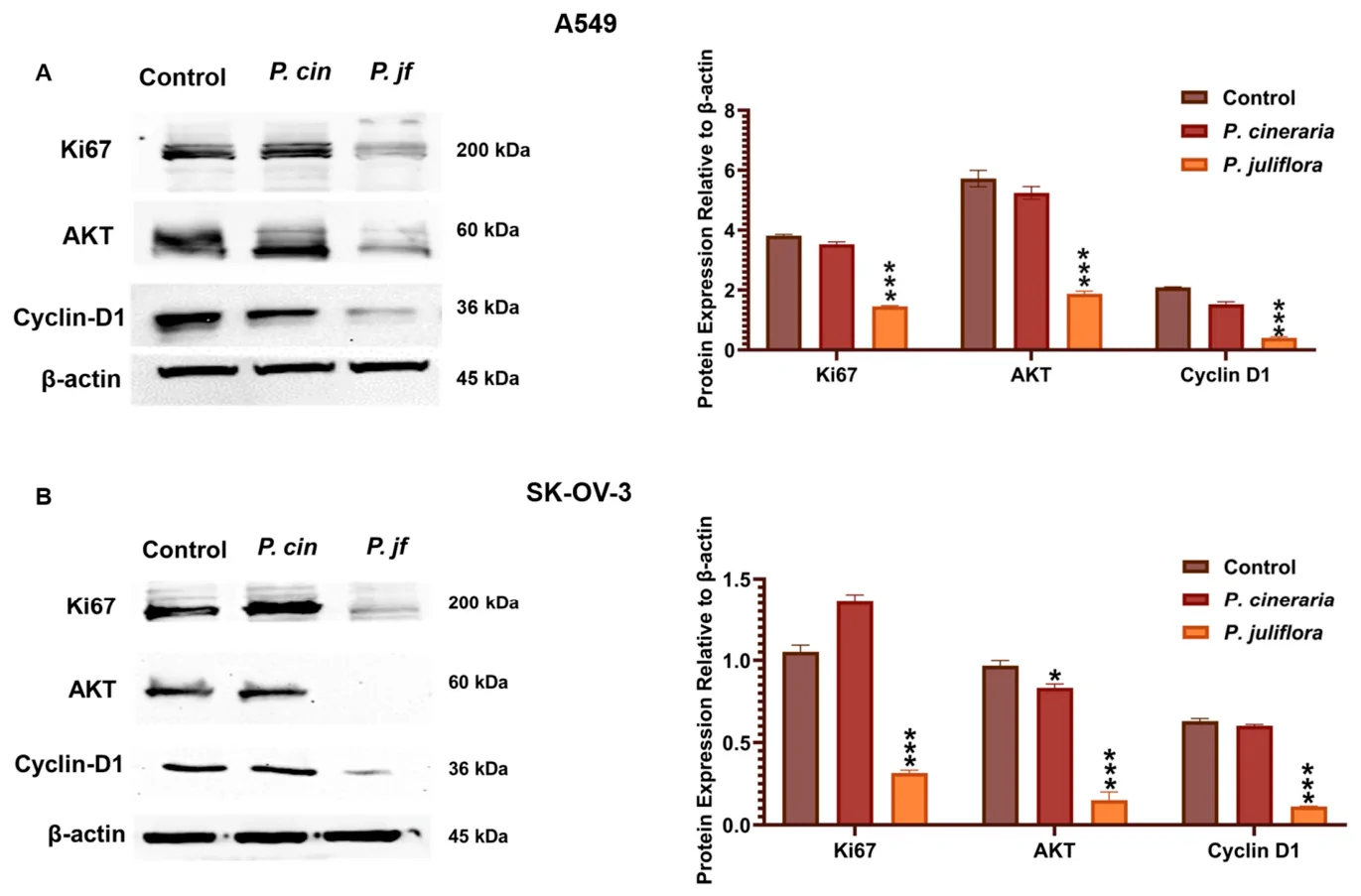
*P. juliflora*, but not *P. cineraria* leaf extracts reduce the levels of proliferation and survival markers in human ovarian and lung cancer cells. (**A**) Western blot analysis of Ki67, AKT, and Cyclin D1 in protein lysates of A549 cells treated with 50 μg/mL of *P. cineraria* (P. cin) or *P. juliflora* (P. jf) extracts. The right panel shows the quantification of the protein bands in the Western blots performed using Image J. Normalization was performed relative to the protein bands of the housekeeping protein β-actin, which served as a loading control. (**B**) Same as in (**A**), but for SK-OV-3 cells. Data are presented as mean ± SEM of three independent experiments (*n* = 3). * indicates *p* < 0.05 and *** indicate *p* < 0.001.

**Figure 7:**
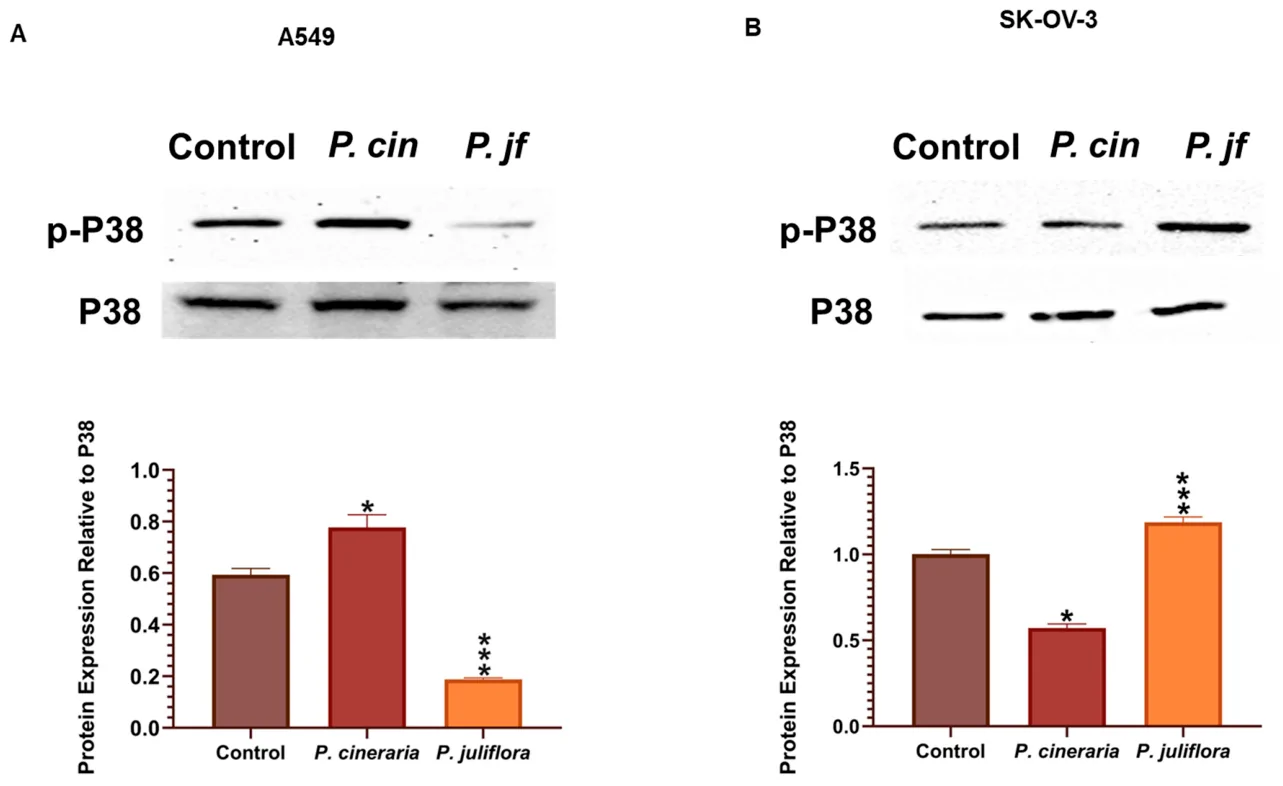
The effect of *P. cineraria* and *P. juliflora* extracts on p38 MAPK activation in A549 and SK-OV-3 cell lines. (**A**) Western blot analysis of p-P38 and P38 in protein lysates of A549 cells treated with 50 μg/mL of *P. cineraria* (P. cin) or *P. juliflora* (P. jf) extracts. The lower panel shows the quantification of the protein bands in the Western blots performed using Image J. Normalization of phosphorylated p38 was performed relative to total p38 protein levels. (**B**) Same as in (**A**), but for SK-OV-3 cells. Data are presented as mean ± SEM of three independent experiments (*n* = 3). * indicates *p* < 0.05 and *** indicate *p* < 0.001.

**Figure 8:**
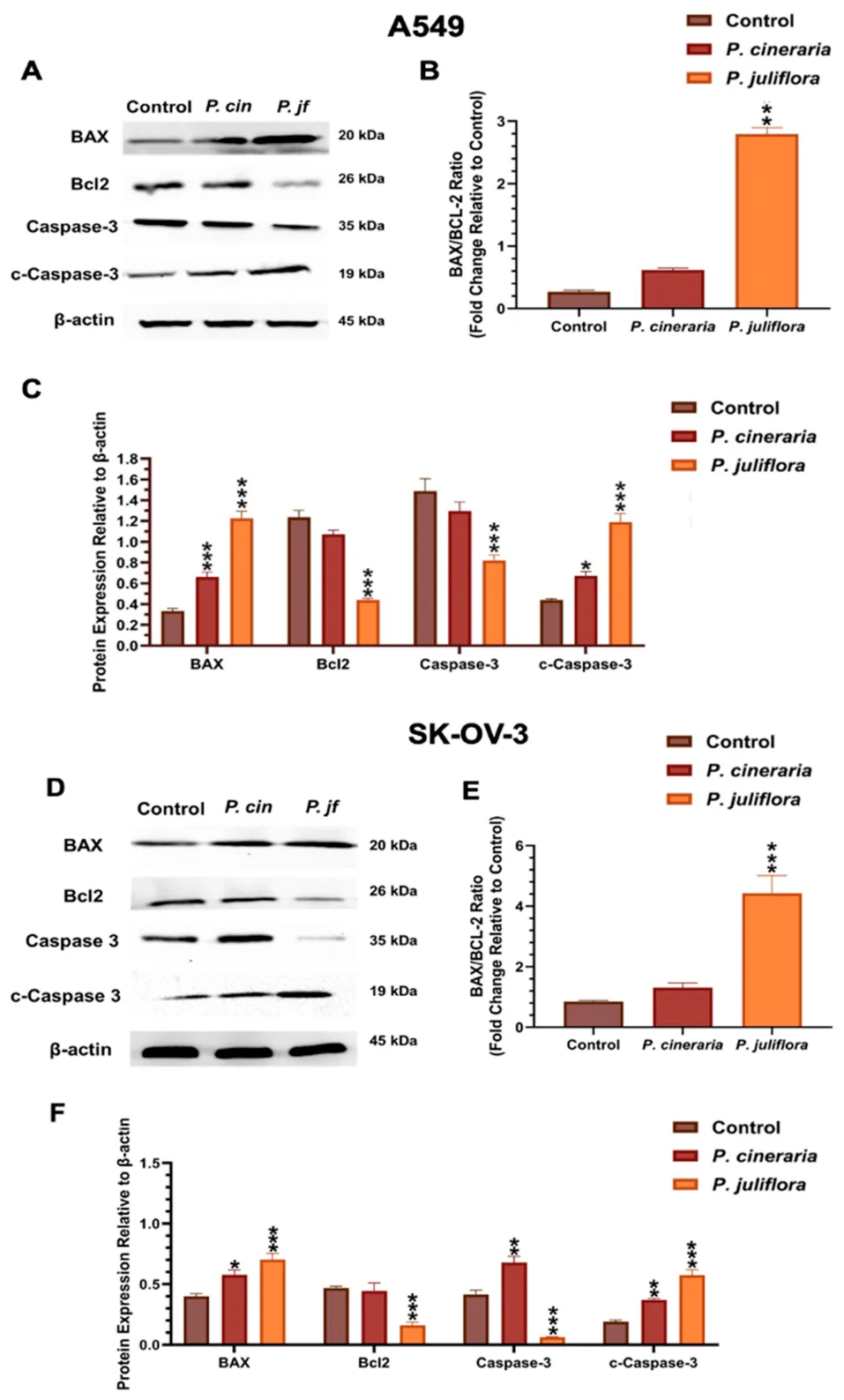
The effect of *P. cineraria* and *P. juliflora* extracts on the expression of apoptosis-related proteins in A549 and SK-OV-3 cell lines. (**A**) Representative Western blots images showing the expression levels of BAX, Bcl-2, Caspase-3 and c-Caspase-3 following treatment with *P. cineraria* (P. Cin) and *P. juliflora* (P. JF) extracts (50 μg/mL) in A549 cells. (**B**) Calculation of the ratio of BAX/ BCL-2 protein levels from (**A**) in A549 cells. (**C**) Quantification of protein expression levels relative to β-actin in A549 cells. (**D**) Same as in (**A**) but for SK-OV-3 cells. (**E**) Same as in (**B**) but for SK-OV-3 cells. (**F**) Same as in (**C**) but for SK-OV-3 cells. The density was analyzed using ImageJ software. Data are presented as mean ± SEM of three independent experiments (*n* = 3). (* *p* < 0.05), (** *p* < 0.01), (*** *p* < 0.001).

**Figure 9:**
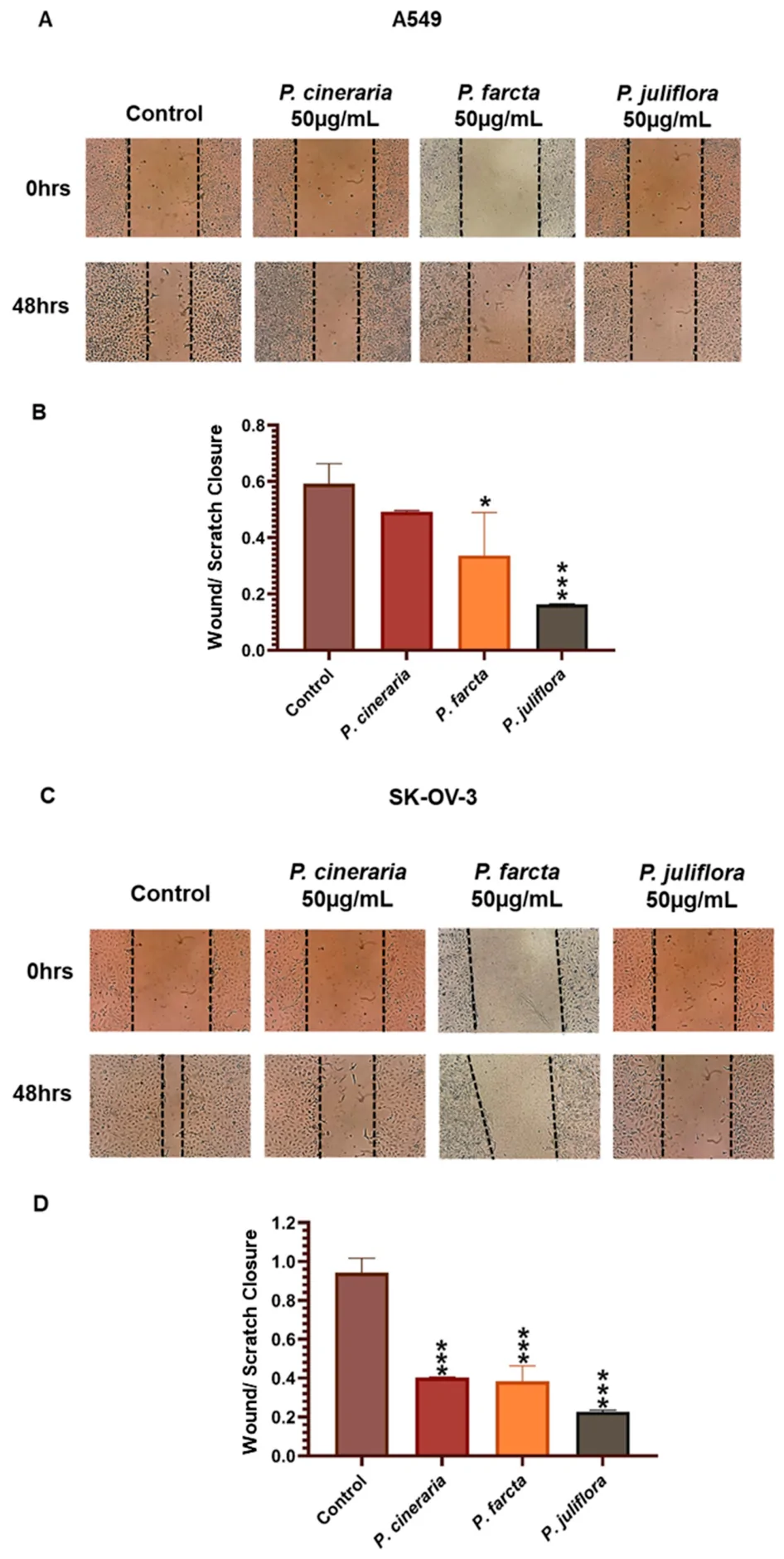
Anti-migratory effects of *P. cineraria*, *P. farcta*, and *P. juliflora* extracts in A549 and SK-OV-3 cells. Confluent monolayers of A549 or SK-OV-3 cells were scratched and treated with the three plant extracts for 48 h. (**A**) Representative images of the scratch area at 0 h and 48 h following scratching in A549 cells. Images were captured at 10× magnification. (**B**) Quantification of wound healing rates. Image J was used to calculate the scratch closure rate. (**C**) Same as in (**A**) but for SK-OV-3 cells. (**D**) Same as in (**A**) but for SK-OV-3 cells. Data are presented as mean ± SEM of three independent experiments (*n* = 3). * indicates *p* < 0.05 and *** indicate *p* < 0.001.

**Table 1:** GC–MS analysis of *P. cineraria*, *P. farcta*, and *P. juliflora* leaf extracts. The identified compounds and their retention times (RT) are presented. The listed phytochemicals were identified by comparing mass fragmentation spectra to those available in the NIST database.

No.	Compounds	RT (min)	*P. cineraria*	*P. farcta*	*P. juliflora*
1	2,3-dihydroinden-1-one (1-Indanone)	3.43	−	−	+
2	4 αH, 5αH-Eudesmane	5.15	−	−	+
3	Caryophyllene	5.73	+	−	+
4	Aromandendrene	6.30	+	−	+
5	Alpha-Curcumene	6.3	−	−	+
6	Humulene	7.33	−	−	+
7	12-Oleanen-3-yl acetate, (3.alpha.)	7.56	+	−	−
8	Caryophyllene oxide	7.62	+	−	+
9	11-Hexadecyn-1-ol	7.95	−	−	+
10	(-)-Spathulenol	8.61	+	−	−
11	Astaxanthin	9.02	−	+	−
12	Ylangene	9.07	−	−	+
13	Nootkatone	9.44	+	+	+
14	Corymbolone	10.79	−	−	+
15	dl-Isopulegol	10.95	−	−	+
16	Barringenol R1	11.75	+	−	−
17	Tetrapentacontane	13.50	−	−	+
18	Chanoclavine	14.10	+	−	+
19	(E)-Limonene oxide	14.93	−	−	+
20	2-Hexadecyloxirane	16.21	−	−	+
21	Octacosane	17.13	−	−	+
22	Z,Z-6,28-Heptatriactontadien-2-one	17.32	−	−	+
23	Blumenol C	17.72	+	−	+
24	alpha-Tocopherol	17.96	+	+	+
25	Campesterol	19.39	+	−	+
26	Hexadecyloxirane	19.45	+	+	+
27	Octacosanol	20.54	−	+	−
28	gamma-Sitosterol	20.90	+	−	+
29	2-Pentatriacontanone	21.17	−	−	+
30	Lanosterol	21.35	+	−	−
31	Lup-20(29)-en-3-one (betulone)	21.58	+	+	+
32	Lupeol	22.56	+	+	+
33	Lup-20(29)-en-3.beta.-ol, acetate	23.40	+	−	+
34	Cholest-4-en-3-one	23.49	+	−	+
35	Pregn-4-ene-3,20-dione,16,17-epoxy, (16.alpha.)-	23.78	+	−	−
36	Betulin	24.62	+	−	−

+: present and −: absent. RT: retention time.

**Table 2:** Selective Index of the cytotoxic effect of *P. juliflora* extract on cancerous versus normal human cells.

	HDFn	A549	SK-OV-3
*P. juliflora* IC50 (µg/mL)	128 ± 5.6	42.2 ± 2.55	49.8 ± 5.34
Selectivity Index (SI)	-	3.03	2.57

## Data Availability

The data are contained within the article. Any additional data can be requested from the corresponding authors.
